# Comparing the Costs of Surveillance of Early-Stage Breast Cancer by Digital or Traditional Follow-Up Methods: Randomized Crossover Study

**DOI:** 10.2196/58113

**Published:** 2025-08-21

**Authors:** Maria Peltola, Carl Blomqvist, Niilo Färkkilä, Paula Poikonen-Saksela, Johanna Mattson

**Affiliations:** 1 Helsinki University Hospital Comprehensive Cancer Center, University of Helsinki, Haartmaninkatu 4, building 2, P.O. Box 180, Helsinki, 00029 HUS, Finland, 358 94711; 2 Amgen Ab, Espoo, Finland

**Keywords:** electronic patient-reported outcome, breast cancer, follow-up costs, surveillance, radiotherapy, randomized study, linear analysis, digital solution, outpatient, communication, cost, mobile app, smartphone, digital app, mHealth, phone calls, follow-up, check-ups, ePRO

## Abstract

**Background:**

An increasing number of early-stage breast cancer (EBC) survivors and limited health care resources have raised interest in developing digital methods for communication between patients and health care personnel. In 2015, Helsinki University Hospital (HUS) Comprehensive Cancer Center (CCC) launched a digital solution called Noona (Helsinki University Hospital; Noona Healthcare) for patients with cancer, which allows patients to report their symptoms or side effects and ask questions with a computer or smart mobile device.

**Objective:**

In this study, we compare the cost and contacts of surveillance of EBC by 2 follow-up methods: digital solution and phone calls during their first year of follow-up outside preplanned visits.

**Methods:**

This was a prospective, open-label, randomized crossover study. After postoperative radiotherapy, patients with EBC were randomized to surveillance with either a digital solution or phone calls in addition to routine follow-up visits. After 6 months, the patient switched to the alternative follow-up method. All patients were thus exposed to both follow-up methods, and the order was determined by randomization. Hospital contacts and the costs of specialized health care were extracted from the Ecomed database of the Helsinki and Uusimaa Hospital District. The Ecomed database records all hospital costs. The costs of follow-up visits and diagnostics at the HUS CCC were analyzed in a repeated measurements general linear model analysis.

**Results:**

The study extended from July 2015 to January 2017. Of 765 patients, 734 were included in the final analyses. For the digital solution group, the mean number of contacts per patient was 1.06 (SD 1.57) during the first 6-month period and 1.22 (SD 1.04) in the second period, with associated costs of €269 (US $313.21) and €311 (US $362.11). Similarly, in the phone call group, the mean number of contacts increased from 0.95 (SD 1.39) to 1.24 (SD 1.14) with the costs of €236 (US $274.78) and €344 (US $400.53), respectively. There were no statistically significant differences in the number of outpatient contacts (*P*=.46 and *P*=.35) or total costs (*P*=.80 and *P*=.12) between the 2 follow-up methods or randomization groups.

**Conclusions:**

We did not find any statistically significant differences in the total cost of follow-up of EBC by digital solution or phone calls. The number of visits and costs were higher during the latter follow-up period, probably due to the scheduled routine 1-year visit. There were more visits and higher costs in the digital solution group during the first 6 months, but these were higher in the phone call group during the latter 6-month period. This shows that the digital solution may enable faster access to outpatient services than conventional follow-up.

## Introduction

Breast cancer is the most common cancer among women. Due to early detection of disease and effective treatments, breast cancer mortality has declined. The 5-year relative overall survival after breast cancer treatment is approximately 90% in Western countries [1,2]. However, breast cancer is still the leading cause of cancer death for women worldwide [3-6].

All breast cancer treatments (chemotherapy, radiotherapy, and endocrine therapy) may cause short- and long-term adverse events. The main aims of breast cancer follow-up are to detect recurrences, give advice, treat long-term side effects, and provide psychological support [5,7-9]. The follow-up protocol must be balanced with regard to the workload on health care, patients’ needs, and costs of follow-up [5,10].

In 2000, Helsinki University Hospital (HUS) Comprehensive Cancer Center (CCC) started a breast cancer nurse practitioner telephone service in addition to regular mammograms and appointments with an oncologist.

Due to the increasing number of breast cancer survivors and limited health care resources, there was a need to develop web-based systems for communication between patients and professionals. In 2015, a web-based patient report app Noona (Helsinki University Hospital; Noona Healthcare) was developed at the HUS CCC in collaboration with a Finnish start-up company. Noona app enabled patients to contact specialist breast cancer nurses between visits. It allows patients to report their cancer- and treatment-related symptoms and health outcomes to the hospital, and to ask questions and deliver messages remotely via a computer, smartphone, or tablet [11].

This prospective controlled randomized crossover study (ClinicalTrials.gov ID NCT04980989) was set up to test the digital solution during the first year of follow-up of early-stage breast cancer (EBC) outside preplanned visits. A total of 765 patients were randomized into sequential digital and conventional phone call follow-up during the first follow-up year, 734 of which were included in this analysis. The order of the 2 follow-up methods was determined by randomization. The results on the primary end point, patient preference, have been previously reported [12]. There was no statistically significant difference in patient preference for the 2 follow-up methods among 142 patients who had experience with both follow-up methods. A total of 40% (56/142) preferred the phone call service and 30% (43/142) the digital solution, while 30% considered both modalities equally good.

The purpose of this study was to analyze whether the cost of follow-up of EBC by a digital solution or phone call differs during the first year outside preplanned visits.

## Methods

### Participants and Follow-Up

Patients with EBC who received radiotherapy after breast cancer surgery were eligible for the study. Inclusion criteria allowed chemotherapy and endocrine therapy if indicated. Patients had to have histologically verified breast cancer or ductal carcinoma in situ, be aged ≥18 years old, and understand Finnish or Swedish. Patients and their tumors’ characteristics were collected from the patient records. Routine follow-up consisted of 3 preplanned appointments at 1, 3, and 5 years after primary diagnosis. Mammography was performed yearly. In addition, patients were requested to contact an educated breast cancer nurse practitioner for symptoms that might be related to breast cancer or its treatment according to a written patient information. Patients were asked to participate in the study on their last appointment with the oncologist at the end of adjuvant radiotherapy. Eligible patients who signed a written consent were included in the study.

The participants were randomized to contact the breast cancer nurse in 2 alternative ways: via the digital solution or the phone call service. After 6 months, the patients switched surveillance methods. The total follow-up time in this study protocol was 1 year. Thus, all patients were exposed to both follow-up methods. When using the digital solution, patients were also allowed to contact the nurse by phone. However, during the phone call follow-up period, patients were not able to use the digital solution.

The purpose of randomization was to ensure that all patients used both monitoring methods and were able to compare them. In addition, we ensured that the differences between the follow-up methods were not due to the order of the 2 methods.

### Costs

The costs of specialized medical care were obtained from the Ecomed database of the Helsinki and Uusimaa Hospital District. Ecomed records contain all hospital resource use and costs including information about the exact day on which the hospital contact started and ended, diagnosis and procedure code, type of contact, and patient-level costs, including overheads, equipment, hospitalization, and drugs for inpatient use. Hospital contacts included hospitalization periods, emergency visits, doctor or nurse appointments, phone calls, letters, and rehabilitation. Contacts to the Department of Oncology of the CCC were classified as contacts due to breast cancer.

Costs of outpatient contacts included outpatient appointments with a doctor or nurse, rehabilitation, medical aids (eg, compression sleeves), letters, and phone calls from hospital personnel to the patient.

The costs of phone calls to the nurse and use of the digital “Noona” solution were free of charge and thus not included in the costs in the analysis. The definition of a letter and phone call is a prearranged individual letter or phone call about the diagnostics and treatment of the patient’s illness, which can replace a visit.

Costs of diagnostics included laboratory tests, pathology reports, and imaging.

### Statistical Analyses

All analyses were carried out using SPSS, version 25 (IBM Corp.). The significance levels were set at *P*<.05.

The effect of the follow-up method was analyzed in repeated measurements general linear model analysis with the follow-up method as a repeated measurement variable and randomization group as a between-patients variable. This design allows testing the effect of both follow-up methods and the effect of timing (first or second 6-month period) on the number of visits and costs. Dependent variables were the number of visits, the costs of diagnostics and visits, and total costs.

Descriptive statistics were calculated for the digital solution and the phone call solution at baseline, at 6 months, and at 12 months.

### Ethical Considerations

This prospective, open-label, and randomized crossover study was registered at the HUS CCC in Finland (ClinicalTrials.gov ID NCT049809899). Written informed consent was obtained from each eligible patient before participation in the trial. The study complied with local ethical and legal requirements. The informed consent and study protocol were approved by the Institutional Ethics Committee of HUS, Finland (trial number Dnro 112/13/03/2015).

## Results

From July 2015 to January 2017, 765 patients with EBC were randomized to this study at the HUS CCC in Finland.

**Figure 1.**
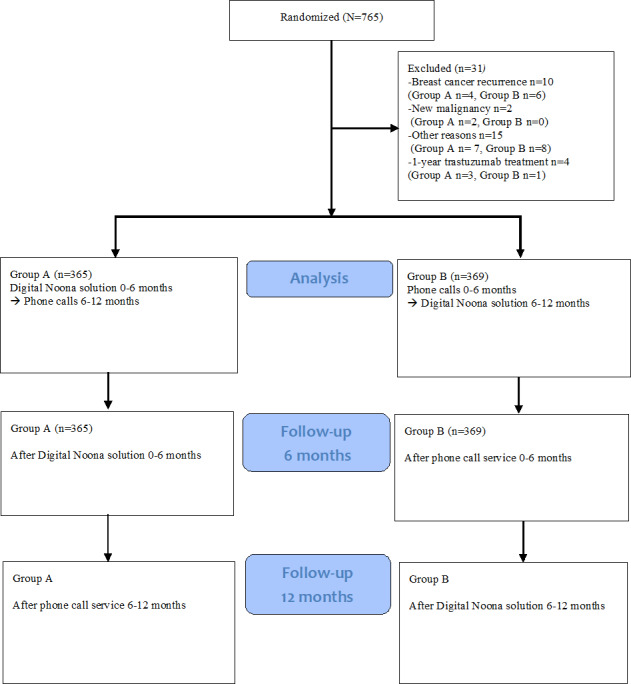
Flowchart of study participants.

A total of 734 patients were included in the final analyses. Two individuals were excluded from the study due to a new malignancy, 10 due to recurrence of breast cancer, and 15 discontinued the study. In addition, 4 patients receiving adjuvant trastuzumab treatment every 3 weeks during the study period were excluded from the analyses, since the costs of follow-up could not be separated from treatment costs in the Ecomed database (Figure 1).

Patient characteristics (n=734) are shown in Table 1. The median age was 61 (range 28‐83) years. Most patients, 630 out of 734, had estrogen receptor–positive breast cancer (85.8%). Two patients out of 734 were male (0.3%).

Hospital contacts during the two 6-month study periods are shown in Table 2. During the 12-month follow-up period, the mean number of hospital contacts was 4.50 (SD 5.14). The mean number of hospital contacts due to breast cancer was 2.23 (SD 1.84). A total of 49% (360/734) of the patients had only one contact, while 29% (213/734) of the patients had 3 or more contacts. There were no hospitalization periods or emergency visits due to breast cancer.

**Table 1.** Patient characteristics.

Characteristics		Group A^a^ (n=365)	Group B^b^ (n=369)	Total (N=734)
Age (years), median (range)		61 (28‐80)	61 (33‐83)	61 (28‐83)
Tumor size, n (%)				
Tis		26 (7.0)	19 (5.2)	45 (6.1)
T1		257 (70.4)	253 (68.6)	510 (69.5)
T2		64 (17.5)	73 (19.8)	137 (18.7)
T3		19 (5.2)	12 (3.3)	31 (4.2)
T4		3 (0.8)	1 (0.3)	4 (0.5)
Tx		3 (0.8)	4 (1.1)	7 (1.0)
Nodal status, n (%)				
N0		212 (58.1)	220 (59.6)	432 (58.9)
N0i+		12 (3.3)	6 (1.6)	18 (2.5)
N1mi		15 (4.1)	18 (4.9)	33 (4.5)
N1		76 (20.8)	78 (21.1)	154 (21.0)
N2		16 (4.4)	17 (4.6)	33 (4.5)
N3		17 (4.7)	11 (3.0)	28 (3.8)
Unknown		17 (4.7)	19 (5.1)	36 (4.9)
Estrogen receptor–positivity, n (%)				
<10		37 (10.2)	24 (6.5)	61 (8.3)
≥10		309 (84.6)	321 (87.0)	630 (85.8)
Not defined		19 (5.2)	24 (6.5)	43 (5.9)
Chemotherapy, n (%)				
Adjuvant		141 (38.6)	138 (37.4)	279 (38.0)
Neoadjuvant		9 (2.5)	6 (1.6)	15 (2.0)
Radiotherapy, n (%)		365 (100)	369 (100)	734 (100)
Endocrine treatment, n (%)		267 (73.2)	267 (72.4)	534 (72.8)

a Digital solution for the first 6 months and phone calls for months 6‐12.

b Phone calls for the first 6 months and digital solution for months 6‐12.

**Table 2.** Hospital contacts per group.

Characteristics		0‐6 months		6‐12 months		Total
		Digital solution	Phone calls	Digital solution	Phone calls	
All hospital contacts, n (share of all contacts, %)		817 (100)	830 (100)	869 (100)	787 (100)	3303 (100)
Hospitalization periods		38 (5)	17 (2)	26 (3)	14 (2)	95 (3)
Emergency visits		42 (5)	22 (3)	15 (2)	19 (2)	98 (3)
Appointments		533 (65)	588 (71)	585 (67)	533 (68)	2239 (68)
Phone calls and letters		191 (23)	188 (23)	240 (28)	215 (27)	834 (25)
Rehabilitation		13 (2)	15 (2)	3 (0)	6 (1)	37 (1)
All contacts per patient						
Mean (SD)		2.24 (3.73)	2.25 (3.63)	2.36 (2.56)	2.16 (2.59)	4.50 (5.14)
Median (IQR)		1 (0‐3)	1 (0‐3)	2 (1-3)	2 (1-3)	3 (1-5)
Hospital contacts due to breast cancer, n (share of all contacts, %)		388 (47)	350 (42)	451 (52)	451 (57)	1640 (50)
Hospitalization periods		0 (0)	0 (0)	0 (0)	0 (0)	0 (0)
Emergency visits		0 (0)	0 (0)	0 (0)	0 (0)	0 (0)
Appointments		252 (31)	233 (28)	296 (34)	298 (38)	1079 (33)
Phone calls and letters		133 (16)	115 (14)	152 (17)	149 (19)	549 (17)
Rehabilitation		3 (0)	2 (0)	3 (0)	4 (1)	12 (0)
All contacts per patient						
Mean (SD)		1.06 (1.56)	0.95 (1.39)	1.22 (1.04)	1.24 (1.14)	2.23 (1.84)
Median (IQR)		0 (0‐2)	0 (0‐2)	1 (1-2)	1 (1-2)	2 (1-3)

The analyses of outpatient contacts and costs by follow-up method and period are shown in Table 3. There were no significant differences between the 2 follow-up methods or randomization arms. However, there was a statistically significant interaction between follow-up method and randomization arm in outpatient contacts, outpatient costs, diagnostics costs, and total costs (*P* values for time: *P*=.001, *P*<.001, *P*=.002, *P*<.001, respectively).

The number of contacts and costs were lower in the first follow-up period compared to the second period. For the digital solution, the mean number of contacts was 1.06 (SD 1.57) in the first period versus 1.22 (SD 1.04) in the second, with costs of €269 (US $313.21) compared to €311 (US $362.11). For the phone call solution, the mean number of contacts increased from 0.95 (SD 1.39) in the first period to 1.24 (SD 1.14) in the second, while costs increased from €236 (US $274.78) to €334 (US $388.89).

In addition, in the first follow-up period, the number of contacts and costs associated with the digital solution were higher than those associated with phone calls, with 1.06 contacts and costs of €269 (US $313.21) for digital versus 0.95 contacts and €236 (US $274.78) for phone calls. However, in the latter period, the costs and contacts using phone calls exceeded those using the digital solution, recording 1.24 contacts and costs of €334 (US $388.89) for phone calls compared to 1.22 contacts and €311 (US $362.11) for the digital solution.

In Table 3, the first *P* value for the follow-up method indicates whether there is an overall difference between the 2 follow-up methods (digital solution or phone calls). The second *P* value for the time period indicates whether the measurement time point (6 or 12 months) affects the difference between the methods. The third *P* value for arm indicates whether the mean score of Group A at 6 and 12 months differs from the mean score of Group B, assessing the difference between the randomization groups (digital solution from baseline to 6 months and phone calls between 6 and 12 months or vice versa).

**Table 3.** Number of outpatient contacts and costs (in €) according to the follow-up method and period or the whole randomization arm.

	Mean per patient (SD)						Statistical significance		
	0‐6 months		6‐12 months		Total		*P* value		
	Digital solution (n=365)	Phone calls (n=369)	Digital solution (n=369)	Phone calls (n=365)	Digital solution (n=734)	Phone calls (n=734)	Follow-up method^a^	Time period^b^	Arm^c^
Outpatient contacts (number)	1.06 (1.57)	0.95 (1.39)	1.22 (1.04)	1.24 (1.14)	1.14 (1.33)	1.09 (1.28)	.46	.001	.35
Outpatient costs (€)^d^	181 (268)	171 (272)	217 (207)	224 (233)	199 (240)	198 (255)	.93	<.001	.53
Diagnostics costs (€)^d^	88 (186)	65 (138)	94 (127)	110 (155)	91 (159)	87 (148)	.64	.002	.01
Total costs (€)^d^	269 (407)	236 (365)	311 (276)	334 (331)	290 (348)	285 (352)	.80	<.001	.12

a Statistical significance in overall difference between the 2 follow-up methods (digital solution or phone calls).

b Statistical significance of the effect of follow-up period (0‐6 or >6 <12 months) was tested in the repeated measurement general linear model as the interaction between follow-up method (digital solution or phone calls) and randomization arm.

c Statistical significance of difference between randomization groups (digital solution from baseline to 6 months and phone calls between 6 and 12 months or vice versa).

d A currency exchange rate of €1=US $1.17 is applicable.

## Discussion

### Principal Findings

The main finding of this study was that the number of hospital contacts and costs during the first follow-up year of patients with EBC with the digital Noona solution did not differ from those of a traditional phone call service. During both surveillance methods, the number of contacts and costs were higher during the latter 6 months of follow-up. This is probably explained by the preplanned routine 1-year visit scheduled approximately 1 year after surgery, which for most patients occurred during the latter study period.

The mean number of hospital contacts at the Department of Oncology was 2.23 (SD 1.84), and the mean number of contacts was 4.50 (SD 5.14), when hospital contacts in specialties other than oncology were included. Thus, breast cancer survivors need frequent medical care both due to cancer and for other reasons during the early part of follow-up. The patients had, for example, gynecological or traumatological visits due to other diseases or accidents.

### Comparison to Prior Work

The average costs of 6 months of surveillance were similar in both groups, that is, €290 (US $337.66) in the digital solution group and €285 (331.84) in the phone call group. Thus, the costs of EBC follow-up were relatively low and in line with previous studies [13,14]. During the first year of follow-up, breast cancer recurrence is rare. Kokko et al [13] showed that the total costs of EBC follow-up over 4.2 years ranged from €1050 (US $1222.55) to €2269 (US $2641.87) per patient (6 months mean €125 [US $145.54] to €270 [US $314.37]), depending on the follow-up method in patients whose disease had not recurred. Another Finnish study also obtained similar results; the mean outpatient cost of EBC follow-up for 6 months was €192 (US $223.55)[14].

Perhaps the most clinically intriguing finding in our study was that diagnostics and costs tended to occur earlier during the follow-up with the digital solution. A possible explanation for this could be that the digital solution provided faster access to the hospital than the phone call service. Results from several previous studies support this interpretation [15,16]. In a previous report of this study on patient preference and satisfaction, satisfaction of patients with EBC was equally high during the digital solution and traditional phone call service. However, patients graded the timeliness of response higher during the digital solution period [12].

Previous studies in metastatic cancer may also support this interpretation. Denis et al [17] randomized 133 patients with metastatic lung cancer (Stage III-IV) after primary treatment to conventional surveillance with scheduled imaging or to use the digital electronic patient-reported outcomes (ePRO) app (Noona Healthcare Oy) once a week. In the ePRO arm, recurrence was detected earlier, fewer computer tomographies were needed, quality of life was better, and median overall survival was 7 months longer [17]. Cost-effectiveness was a secondary end point of the study, and annual cost in the ePRO arm was €362 (US $421.49) lower per patient than in the conventional arm [18].

To our knowledge, this is the first health economy study comparing ePRO and conventional follow-up in EBC. The strengths of our study are the large sample size and the high compliance rate. The characteristics of the patients were well representative and similar to previous studies in patients with EBC [5,19]. The costs of the follow-up were obtained directly from the hospital Ecomed database.

### Limitations

We are aware of the limitations of the study. First, the study included a short follow-up time of 12 months, so long-term results could not be evaluated. Second, patients with relapsed disease were excluded from the study because of the crossover study design. Third, it is not possible to report the proportion of additional phone contacts during the digital solution period. Fourth, the data were collected from July 2015 to January 2017, and costs may have changed. Since the COVID-19 pandemic, the need for remote oncology services has expanded, and the acceptability of using digital solutions may have increased. As the costs were the same, this should not have had an impact on our results.

### Future Directions

In summary, we did not find any statistically significant differences in the total cost of follow-up of EBC by digital solution or phone calls. More diagnostic tests and visits occurred during the first half of the follow-up period in the digital solution group, indicating that digital follow-up may enable faster access to outpatient services than conventional follow-up. There is a need to develop digital services for the follow-up of breast cancer.

### Conclusions

We did not find any statistically significant differences in the total cost of follow-up of EBC by digital solution or phone calls. The number of visits and costs were higher during the latter follow-up period, probably due to the scheduled routine 1-year visit. There were more visits, and costs were higher in the digital solution group during the first 6 months, but higher in the phone call group during the latter 6-month period. This shows that the digital solution may enable a faster access to outpatient services than conventional follow-up.

## Supplementary material

10.2196/58113 Checklist 1 CONSORT-eHEALTH checklist (V 1.6.1). CONSORT: Consolidated Standards of Reporting Trials.

## Abbreviations

CCC: Comprehensive Cancer Center
EBC: early-stage breast cancer
ePRO: electronic patient-reported outcome
HUS: Helsinki University Hospital
