# Supplementary material for: Comparing the Costs of Surveillance of Early-Stage Breast Cancer by Digital or Traditional Follow-Up Methods: Randomized Crossover Study
Source: JMIR Cancer. 2025 Aug 21;11:e58113. doi: 10.2196/58113 (PMC12369445; doi:10.2196/58113)
Supplement: Checklist 1 [file cancer-v11-e58113-s001.pdf]

CONSORT-EHEALTH (V 1.6.1) - Submission/Publication Form

The CONSORT-EHEALTH checklist is intended for authors of randomized trials evaluating web-based and Internet-based applications/interventions, including mobile interventions, electronic games (incl multiplayer games), social media, certain telehealth applications, and other interactive and/or networked electronic applications. Some of the items (e.g. all subitems under item 5 - description of the intervention) may also be applicable for other study designs.

The goal of the CONSORT EHEALTH checklist and guideline is to be
a) a guide for reporting for authors of RCTs,
b) to form a basis for appraisal of an ehealth trial (in terms of validity)

CONSORT-EHEALTH items/subitems are MANDATORY reporting items for studies published in the Journal of Medical Internet Research and other journals / scientific societies endorsing the checklist.

Items numbered 1., 2., 3., 4a., 4b etc are original CONSORT or CONSORT-NPT (non-pharmacologic treatment) items.
Items with Roman numerals (i., ii, iii, iv etc.) are CONSORT-EHEALTH extensions/clarifications.

As the CONSORT-EHEALTH checklist is still considered in a formative stage, we would ask that you also RATE ON A SCALE OF 1-5 how important/useful you feel each item is FOR THE PURPOSE OF THE CHECKLIST and reporting guideline (optional).

Mandatory reporting items are marked with a red *.
In the textboxes, either copy & paste the relevant sections from your manuscript into this form - please include any quotes from your manuscript in QUOTATION MARKS,
or answer directly by providing additional information not in the manuscript, or elaborating on why the item was not relevant for this study.

YOUR ANSWERS WILL BE PUBLISHED AS A SUPPLEMENTARY FILE TO YOUR PUBLICATION IN JMIR AND ARE CONSIDERED PART OF YOUR PUBLICATION (IF ACCEPTED).
Please fill in these questions diligently. Information will not be copyedited, so please use proper spelling and grammar, use correct capitalization, and avoid abbreviations.

DO NOT FORGET TO SAVE AS PDF _AND_ CLICK THE SUBMIT BUTTON SO YOUR ANSWERS ARE IN OUR DATABASE !!!

Citation Suggestion (if you append the pdf as Appendix we suggest to cite this paper in the caption):
Eysenbach G, CONSORT-EHEALTH Group

Olet muokkaamassa vastaustasi. Jos jaat tämän URL-osoitteen, myös muut voivat muokata vastaustasi.

KIRJOITA UUSI VASTAUS

URL: http://www.jmir.org/2011/4/e126/
doi: 10.2196/jmir.1923
PMID: 22209829

ptpoikonen@gmail.com Vaihda tiliä

Ei jaettu

Tallenna lähettämällä uudelleen

* Pakollinen kysymys

Your name *

First Last

Paula Poikonen-Saksela

Primary Affiliation (short), City, Country *

University of Toronto, Toronto, Canada

Finland

Your e-mail address *

abc@gmail.com

paula.poikonen-saksela@hus.fi

Title of your manuscript *

Provide the (draft) title of your manuscript.

Comparing the costs of surveillance of early breast cancer by digital or traditional follow-up methods: a randomized cross-over study

Olet muokkaamassa vastaustasi. Jos jaat tämän URL-osoitteen, myös muut voivat muokata vastaustasi.

KIRJOITA UUSI VASTAUS

Name of your App/Software/Intervention *

If there is a short and a long/alternate name, write the short name first and add the long name in brackets.

Noona

Evaluated Version (if any)

e.g. "V1", "Release 2017-03-01", "Version 2.0.27913"

Oma vastauksesi

Language(s) *

What language is the intervention/app in? If multiple languages are available, separate by comma (e.g. "English, French")

Finnish, Swedish, English

URL of your Intervention Website or App

e.g. a direct link to the mobile app on app in appstore (itunes, Google Play), or URL of the website. If the intervention is a DVD or hardware, you can also link to an Amazon page.

Oma vastauksesi

URL of an image/screenshot (optional)

Oma vastauksesi

Olet muokkaamassa vastaustasi. Jos jaat tämän URL-osoitteen, myös muut voivat muokata vastaustasi.

KIRJOITA UUSI VASTAUS

Accessibility *

Can an enduser access the intervention presently?

- ◯ access is free and open
- ◉ access only for special usergroups, not open
- ◯ access is open to everyone, but requires payment/subscription/in-app purchases
- ◯ app/intervention no longer accessible
- ◯ Muu:

Primary Medical Indication/Disease/Condition *

e.g. "Stress", "Diabetes", or define the target group in brackets after the condition, e.g. "Autism (Parents of children with)", "Alzheimers (Informal Caregivers of)"

Cancer

Primary Outcomes measured in trial *

comma-separated list of primary outcomes reported in the trial

patient preference, cost effectivity

Secondary/other outcomes

Are there any other outcomes the intervention is expected to affect?

Oma vastauksesi

Olet muokkaamassa vastaustasi. Jos jaat tämän URL-osoitteen, myös muut voivat muokata vastaustasi.

KIRJOITA UUSI VASTAUS

Recommended "Dose" *

What do the instructions for users say on how often the app should be used?

- ( ) Approximately Daily
- ( ) Approximately Weekly
- ( ) Approximately Monthly
- ( ) Approximately Yearly
- (x) "as needed"
- ( ) Muu:

Approx. Percentage of Users (starters) still using the app as recommended after 3 months *

- ( ) unknown / not evaluated
- ( ) 0-10%
- (x) 11-20%
- ( ) 21-30%
- ( ) 31-40%
- ( ) 41-50%
- ( ) 51-60%
- ( ) 61-70%
- ( ) 71%-80%
- ( ) 81-90%
- ( ) 91-100%
- ( ) Muu:

Olet muokkaamassa vastaustasi. Jos jaat tämän URL-osoitteen, myös muut voivat muokata vastaustasi.

KIRJOITA UUSI VASTAUS

Overall, was the app/intervention effective? *

- ( ) yes: all primary outcomes were significantly better in intervention group vs control
- ( ) partly: SOME primary outcomes were significantly better in intervention group vs control
- (x) no statistically significant difference between control and intervention
- ( ) potentially harmful: control was significantly better than intervention in one or more outcomes
- ( ) inconclusive: more research is needed
- ( ) Muu:

Article Preparation Status/Stage *

At which stage in your article preparation are you currently (at the time you fill in this form)

- ( ) not submitted yet - in early draft status
- ( ) not submitted yet - in late draft status, just before submission
- ( ) submitted to a journal but not reviewed yet
- ( ) submitted to a journal and after receiving initial reviewer comments
- (x) submitted to a journal and accepted, but not published yet
- ( ) published
- ( ) Muu:

Olet muokkaamassa vastaustasi. Jos jaat tämän URL-osoitteen, myös muut voivat muokata vastaustasi.

KIRJOITA UUSI VASTAUS

Journal *

If you already know where you will submit this paper (or if it is already submitted), please provide the journal name (if it is not JMIR, provide the journal name under "other")

- ○ not submitted yet / unclear where I will submit this
- ○ Journal of Medical Internet Research (JMIR)
- ○ JMIR mHealth and UHealth
- ○ JMIR Serious Games
- ○ JMIR Mental Health
- ○ JMIR Public Health
- ○ JMIR Formative Research
- ○ Other JMIR sister journal
- ◉ Muu: JMIR Cancer

Is this a full powered effectiveness trial or a pilot/feasibility trial? *

- ○ Pilot/feasibility
- ◉ Fully powered

Manuscript tracking number *

If this is a JMIR submission, please provide the manuscript tracking number under "other" (The ms tracking number can be found in the submission acknowledgement email, or when you login as author in JMIR. If the paper is already published in JMIR, then the ms tracking number is the four-digit number at the end of the DOI, to be found at the bottom of each published article in JMIR)

- ○ no ms number (yet) / not (yet) submitted to / published in JMIR
- ◉ Muu: 58113

Olet muokkaamassa vastaustasi. Jos jaat tämän URL-osoitteen, myös muut voivat muokata vastaustasi. KIRJOITA UUSI VASTAUS

TITLE AND ABSTRACT

1a) TITLE: Identification as a randomized trial in the title

1a) Does your paper address CONSORT item 1a? *

I.e does the title contain the phrase "Randomized Controlled Trial"? (if not, explain the reason under "other")

- ( ) yes
- (x) Muu: title contains phrase randomized croos over study

1a-i) Identify the mode of delivery in the title

Identify the mode of delivery. Preferably use “web-based” and/or “mobile” and/or “electronic game” in the title. Avoid ambiguous terms like “online”, “virtual”, “interactive”. Use “Internet-based” only if Intervention includes non-web-based Internet components (e.g. email), use “computer-based” or “electronic” only if offline products are used. Use “virtual” only in the context of “virtual reality” (3-D worlds). Use “online” only in the context of “online support groups”. Complement or substitute product names with broader terms for the class of products (such as “mobile” or “smart phone” instead of “iphone”), especially if the application runs on different platforms.

subitem not at all important

- 1 ( )
- 2 ( )
- 3 ( )
- 4 ( )
- 5 ( )

essential

Olet muokkaamassa vastaustasi. Jos jaat tämän URL-osoitteen, myös muut voivat muokata vastaustasi.

KIRJOITA UUSI VASTAUS

Does your paper address subitem 1a-i? *

Copy and paste relevant sections from manuscript title (include quotes in quotation marks "like this" to indicate direct quotes from your manuscript), or elaborate on this item by providing additional information not in the ms, or briefly explain why the item is not applicable/relevant for your study

Our title is : Comparing the costs of surveillance of early breast cancer by digital or traditional follow-up methods: a randomized cross-over study

1a-ii) Non-web-based components or important co-interventions in title

Mention non-web-based components or important co-interventions in title, if any (e.g., “with telephone support”).

subitem not at all important

1 ○

2 ○

3 ○

4 ○

5 ○

essential

Does your paper address subitem 1a-ii?

Copy and paste relevant sections from manuscript title (include quotes in quotation marks "like this" to indicate direct quotes from your manuscript), or elaborate on this item by providing additional information not in the ms, or briefly explain why the item is not applicable/relevant for your study

Oma vastauksesi

Olet muokkaamassa vastaustasi. Jos jaat tämän URL-osoitteen, myös muut voivat muokata vastaustasi.

KIRJOITA UUSI VASTAUS

1a-iii) Primary condition or target group in the title

Mention primary condition or target group in the title, if any (e.g., “for children with Type I Diabetes”) Example: A Web-based and Mobile Intervention with Telephone Support for Children with Type I Diabetes: Randomized Controlled Trial

subitem not at all important

1 ○

2 ○

3 ○

4 ○

5 ○

essential

Does your paper address subitem 1a-iii? *

Copy and paste relevant sections from manuscript title (include quotes in quotation marks "like this" to indicate direct quotes from your manuscript), or elaborate on this item by providing additional information not in the ms, or briefly explain why the item is not applicable/relevant for your study

Yes , "early breast cancer"

1b) ABSTRACT: Structured summary of trial design, methods, results, and conclusions

NPT extension: Description of experimental treatment, comparator, care providers, centers, and blinding status.

Olet muokkaamassa vastaustasi. Jos jaat tämän URL-osoitteen, myös muut voivat muokata vastaustasi.

KIRJOITA UUSI VASTAUS

1b-i) Key features/functionalities/components of the intervention and comparator in the METHODS section of the ABSTRACT

Mention key features/functionalities/components of the intervention and comparator in the abstract. If possible, also mention theories and principles used for designing the site. Keep in mind the needs of systematic reviewers and indexers by including important synonyms. (Note: Only report in the abstract what the main paper is reporting. If this information is missing from the main body of text, consider adding it)

subitem not at all important

1 ○

2 ○

3 ○

4 ○

5 ○

essential

Does your paper address subitem 1b-i? *

Copy and paste relevant sections from the manuscript abstract (include quotes in quotation marks "like this" to indicate direct quotes from your manuscript), or elaborate on this item by providing additional information not in the ms, or briefly explain why the item is not applicable/relevant for your study

Yes, "After postoperative radiotherapy patients with EBC were randomized to surveillance with either a digital solution or phone calls in addition to routine follow-up visits. After six months the patient switched to the alternative follow-up method. All patients were thus exposed to both follow-up methods and the order was determined by randomization. "

Olet muokkaamassa vastaustasi. Jos jaat tämän URL-osoitteen, myös muut voivat muokata vastaustasi.

KIRJOITA UUSI VASTAUS

1b-ii) Level of human involvement in the METHODS section of the ABSTRACT

Clarify the level of human involvement in the abstract, e.g., use phrases like “fully automated” vs. “therapist/nurse/care provider/physician-assisted” (mention number and expertise of providers involved, if any). (Note: Only report in the abstract what the main paper is reporting. If this information is missing from the main body of text, consider adding it)

subitem not at all important

1 ○

2 ○

3 ○

4 ○

5 ○

essential

Does your paper address subitem 1b-ii?

Copy and paste relevant sections from the manuscript abstract (include quotes in quotation marks "like this" to indicate direct quotes from your manuscript), or elaborate on this item by providing additional information not in the ms, or briefly explain why the item is not applicable/relevant for your study

Oma vastauksesi

Olet muokkaamassa vastaustasi. Jos jaat tämän URL-osoitteen, myös muut voivat muokata vastaustasi.

KIRJOITA UUSI VASTAUS

1b-iii) Open vs. closed, web-based (self-assessment) vs. face-to-face assessments in the METHODS section of the ABSTRACT

Mention how participants were recruited (online vs. offline), e.g., from an open access website or from a clinic or a closed online user group (closed usergroup trial), and clarify if this was a purely web-based trial, or there were face-to-face components (as part of the intervention or for assessment). Clearly say if outcomes were self-assessed through questionnaires (as common in web-based trials). Note: In traditional offline trials, an open trial (open-label trial) is a type of clinical trial in which both the researchers and participants know which treatment is being administered. To avoid confusion, use “blinded” or “unblinded” to indicated the level of blinding instead of “open”, as “open” in web-based trials usually refers to “open access” (i.e. participants can self-enrol). (Note: Only report in the abstract what the main paper is reporting. If this information is missing from the main body of text, consider adding it)

subitem not at all important

1 ○

2 ○

3 ○

4 ○

5 ○

essential

Does your paper address subitem 1b-iii?

Copy and paste relevant sections from the manuscript abstract (include quotes in quotation marks "like this" to indicate direct quotes from your manuscript), or elaborate on this item by providing additional information not in the ms, or briefly explain why the item is not applicable/relevant for your study

Oma vastauksesi

Olet muokkaamassa vastaustasi. Jos jaat tämän URL-osoitteen, myös muut voivat muokata vastaustasi.

KIRJOITA UUSI VASTAUS

1b-iv) RESULTS section in abstract must contain use data

Report number of participants enrolled/assessed in each group, the use/uptake of the intervention (e.g., attrition/adherence metrics, use over time, number of logins etc.), in addition to primary/secondary outcomes. (Note: Only report in the abstract what the main paper is reporting. If this information is missing from the main body of text, consider adding it)

subitem not at all important

1

2

3

4

5

essential

Does your paper address subitem 1b-iv?

Copy and paste relevant sections from the manuscript abstract (include quotes in quotation marks "like this" to indicate direct quotes from your manuscript), or elaborate on this item by providing additional information not in the ms, or briefly explain why the item is not applicable/relevant for your study

Oma vastauksesi

Olet muokkaamassa vastaustasi. Jos jaat tämän URL-osoitteen, myös muut voivat muokata vastaustasi.

KIRJOITA UUSI VASTAUS

1b-v) CONCLUSIONS/DISCUSSION in abstract for negative trials

Conclusions/Discussions in abstract for negative trials: Discuss the primary outcome - if the trial is negative (primary outcome not changed), and the intervention was not used, discuss whether negative results are attributable to lack of uptake and discuss reasons. (Note: Only report in the abstract what the main paper is reporting. If this information is missing from the main body of text, consider adding it)

subitem not at all important

1 ○

2 ○

3 ○

4 ○

5 ○

essential

Does your paper address subitem 1b-v?

Copy and paste relevant sections from the manuscript abstract (include quotes in quotation marks "like this" to indicate direct quotes from your manuscript), or elaborate on this item by providing additional information not in the ms, or briefly explain why the item is not applicable/relevant for your study

Oma vastauksesi

INTRODUCTION

2a) In INTRODUCTION: Scientific background and explanation of rationale

Olet muokkaamassa vastaustasi. Jos jaat tämän URL-osoitteen, myös muut voivat muokata vastaustasi.

KIRJOITA UUSI VASTAUS

2a-i) Problem and the type of system/solution

Describe the problem and the type of system/solution that is object of the study: intended as stand-alone intervention vs. incorporated in broader health care program? Intended for a particular patient population? Goals of the intervention, e.g., being more cost-effective to other interventions, replace or complement other solutions? (Note: Details about the intervention are provided in “Methods” under 5)

subitem not at all important

1 ◯

2 ◯

3 ◯

4 ◯

5 ◯

essential

Does your paper address subitem 2a-i? *

Copy and paste relevant sections from the manuscript (include quotes in quotation marks "like this" to indicate direct quotes from your manuscript), or elaborate on this item by providing additional information not in the ms, or briefly explain why the item is not applicable/relevant for your study

Yes, "Due to the increasing number of breast cancer survivors and limited healthcare resources, there was a need to develop web-based systems for communication between patients and professionals. In 2015, web-based patient report application Noona was developed at the HUS CCC in collaboration with a Finnish start-up company. Noona application enabled patients to contact specialist breast cancer nurses between visits. It allows patients to report their cancer and treatment-related symptoms and health outcomes to the hospital, and ask questions and deliver messages remotely via a computer, smartphone, or tablet. [11]. This prospective controlled randomized cross-over study (ClinicalTrials.gov number: NCT04980989) was set up to test the digital solution during the first year of follow-up of early breast cancer (EBC) outside pre-planned visits."

Olet muokkaamassa vastaustasi. Jos jaat tämän URL-osoitteen, myös muut voivat muokata vastaustasi.

KIRJOITA UUSI VASTAUS

2a-ii) Scientific background, rationale: What is known about the (type of) system

Scientific background, rationale: What is known about the (type of) system that is the object of the study (be sure to discuss the use of similar systems for other conditions/diagnoses, if appropiate), motivation for the study, i.e. what are the reasons for and what is the context for this specific study, from which stakeholder viewpoint is the study performed, potential impact of findings [2]. Briefly justify the choice of the comparator.

subitem not at all important

1 ○

2 ○

3 ○

4 ○

5 ○

essential

Does your paper address subitem 2a-ii? *

Copy and paste relevant sections from the manuscript (include quotes in quotation marks "like this" to indicate direct quotes from your manuscript), or elaborate on this item by providing additional information not in the ms, or briefly explain why the item is not applicable/relevant for your study

Yes, "This prospective controlled randomized cross-over study (ClinicalTrials.gov number: NCT04980989) was set up to test the digital solution during the first year of follow-up of early breast cancer (EBC) outside pre-planned visits. The purpose of the present study was to analyze whether the cost of follow-up of EBC by a digital solution or phone call differ during the first year outside pre-planned visits. "

2b) In INTRODUCTION: Specific objectives or hypotheses

Olet muokkaamassa vastaustasi. Jos jaat tämän URL-osoitteen, myös muut voivat muokata vastaustasi. KIRJOITA UUSI VASTAUS

Does your paper address CONSORT subitem 2b? *

Copy and paste relevant sections from the manuscript (include quotes in quotation marks "like this" to indicate direct quotes from your manuscript), or elaborate on this item by providing additional information not in the ms, or briefly explain why the item is not applicable/relevant for your study

Yes, "The purpose of the present study was to analyze whether the cost of follow-up of EBC by a digital solution or phone call differ during the first year outside pre-planned visits."

METHODS

3a) Description of trial design (such as parallel, factorial) including allocation ratio

Does your paper address CONSORT subitem 3a? *

Copy and paste relevant sections from the manuscript (include quotes in quotation marks "like this" to indicate direct quotes from your manuscript), or elaborate on this item by providing additional information not in the ms, or briefly explain why the item is not applicable/relevant for your study

Yes, "a randomized cross-over study"

3b) Important changes to methods after trial commencement (such as eligibility criteria), with reasons

Does your paper address CONSORT subitem 3b? *

Copy and paste relevant sections from the manuscript (include quotes in quotation marks "like this" to indicate direct quotes from your manuscript), or elaborate on this item by providing additional information not in the ms, or briefly explain why the item is not applicable/relevant for your study

No major fixes was done at the time of this trial, after the trial technical development has been done

Olet muokkaamassa vastaustasi. Jos jaat tämän URL-osoitteen, myös muut voivat muokata vastaustasi.

KIRJOITA UUSI VASTAUS

3b-i) Bug fixes, Downtimes, Content Changes

Bug fixes, Downtimes, Content Changes: ehealth systems are often dynamic systems. A description of changes to methods therefore also includes important changes made on the intervention or comparator during the trial (e.g., major bug fixes or changes in the functionality or content) (5-iii) and other “unexpected events” that may have influenced study design such as staff changes, system failures/downtimes, etc. [2].

subitem not at all important

1 ○

2 ○

3 ○

4 ○

5 ○

essential

Does your paper address subitem 3b-i?

Copy and paste relevant sections from the manuscript (include quotes in quotation marks "like this" to indicate direct quotes from your manuscript), or elaborate on this item by providing additional information not in the ms, or briefly explain why the item is not applicable/relevant for your study

Oma vastauksesi

4a) Eligibility criteria for participants

Does your paper address CONSORT subitem 4a? *

Copy and paste relevant sections from the manuscript (include quotes in quotation marks "like this" to indicate direct quotes from your manuscript), or elaborate on this item by providing additional information not in the ms, or briefly explain why the item is not applicable/relevant for your study

Olet muokkaamassa vastaustasi. Jos jaat tämän URL-osoitteen, myös muut voivat muokata vastaustasi.

KIRJOITA UUSI VASTAUS

4a-i) Computer / Internet literacy

Computer / Internet literacy is often an implicit “de facto” eligibility criterion - this should be explicitly clarified.

subitem not at all important

1 ◯

2 ◯

3 ◯

4 ◯

5 ◯

essential

Does your paper address subitem 4a-i?

Copy and paste relevant sections from the manuscript (include quotes in quotation marks "like this" to indicate direct quotes from your manuscript), or elaborate on this item by providing additional information not in the ms, or briefly explain why the item is not applicable/relevant for your study

Oma vastauksesi

Olet muokkaamassa vastaustasi. Jos jaat tämän URL-osoitteen, myös muut voivat muokata vastaustasi.

KIRJOITA UUSI VASTAUS

4a-ii) Open vs. closed, web-based vs. face-to-face assessments:

Open vs. closed, web-based vs. face-to-face assessments: Mention how participants were recruited (online vs. offline), e.g., from an open access website or from a clinic, and clarify if this was a purely web-based trial, or there were face-to-face components (as part of the intervention or for assessment), i.e., to what degree got the study team to know the participant. In online-only trials, clarify if participants were quasi-anonymous and whether having multiple identities was possible or whether technical or logistical measures (e.g., cookies, email confirmation, phone calls) were used to detect/prevent these.

subitem not at all important

1 ○

2 ○

3 ○

4 ○

5 ○

essential

Does your paper address subitem 4a-ii? *

Copy and paste relevant sections from the manuscript (include quotes in quotation marks "like this" to indicate direct quotes from your manuscript), or elaborate on this item by providing additional information not in the ms, or briefly explain why the item is not applicable/relevant for your study

Yes, face to face meeting

Olet muokkaamassa vastaustasi. Jos jaat tämän URL-osoitteen, myös muut voivat muokata vastaustasi. KIRJOITA UUSI VASTAUS

4a-iii) Information giving during recruitment

Information given during recruitment. Specify how participants were briefed for recruitment and in the informed consent procedures (e.g., publish the informed consent documentation as appendix, see also item X26), as this information may have an effect on user self-selection, user expectation and may also bias results.

subitem not at all important

1 ○

2 ○

3 ○

4 ○

5 ○

essential

Does your paper address subitem 4a-iii?

Copy and paste relevant sections from the manuscript (include quotes in quotation marks "like this" to indicate direct quotes from your manuscript), or elaborate on this item by providing additional information not in the ms, or briefly explain why the item is not applicable/relevant for your study

Oma vastauksesi

4b) Settings and locations where the data were collected

Does your paper address CONSORT subitem 4b? *

Copy and paste relevant sections from the manuscript (include quotes in quotation marks "like this" to indicate direct quotes from your manuscript), or elaborate on this item by providing additional information not in the ms, or briefly explain why the item is not applicable/relevant for your study

Yes, this was adressed see below

Olet muokkaamassa vastaustasi. Jos jaat tämän URL-osoitteen, myös muut voivat muokata vastaustasi. KIRJOITA UUSI VASTAUS

4b-i) Report if outcomes were (self-)assessed through online questionnaires

Clearly report if outcomes were (self-)assessed through online questionnaires (as common in web-based trials) or otherwise.

subitem not at all important

1 ○

2 ○

3 ○

4 ○

5 ○

essential

Does your paper address subitem 4b-i? *

Copy and paste relevant sections from the manuscript (include quotes in quotation marks "like this" to indicate direct quotes from your manuscript), or elaborate on this item by providing additional information not in the ms, or briefly explain why the item is not applicable/relevant for your study

Yes, For primary endpoints patients answered by paper, cost information was collected from electronic registeries

Olet muokkaamassa vastaustasi. Jos jaat tämän URL-osoitteen, myös muut voivat muokata vastaustasi.

KIRJOITA UUSI VASTAUS

4b-ii) Report how institutional affiliations are displayed

Report how institutional affiliations are displayed to potential participants [on ehealth media], as affiliations with prestigious hospitals or universities may affect volunteer rates, use, and reactions with regards to an intervention.(Not a required item – describe only if this may bias results)

subitem not at all important

1 ◯

2 ◯

3 ◯

4 ◯

5 ◯

essential

Does your paper address subitem 4b-ii?

Copy and paste relevant sections from the manuscript (include quotes in quotation marks "like this" to indicate direct quotes from your manuscript), or elaborate on this item by providing additional information not in the ms, or briefly explain why the item is not applicable/relevant for your study

Oma vastauksesi

5) The interventions for each group with sufficient details to allow replication, including how and when they were actually administered

Olet muokkaamassa vastaustasi. Jos jaat tämän URL-osoitteen, myös muut voivat muokata vastaustasi.

KIRJOITA UUSI VASTAUS

5-i) Mention names, credential, affiliations of the developers, sponsors, and owners

Mention names, credential, affiliations of the developers, sponsors, and owners [6] (if authors/evaluators are owners or developer of the software, this needs to be declared in a “Conflict of interest” section or mentioned elsewhere in the manuscript).

subitem not at all important

1 ○

2 ○

3 ○

4 ○

5 ○

essential

Does your paper address subitem 5-i?

Copy and paste relevant sections from the manuscript (include quotes in quotation marks "like this" to indicate direct quotes from your manuscript), or elaborate on this item by providing additional information not in the ms, or briefly explain why the item is not applicable/relevant for your study

Oma vastauksesi

Olet muokkaamassa vastaustasi. Jos jaat tämän URL-osoitteen, myös muut voivat muokata vastaustasi.

KIRJOITA UUSI VASTAUS

5-ii) Describe the history/development process

Describe the history/development process of the application and previous formative evaluations (e.g., focus groups, usability testing), as these will have an impact on adoption/use rates and help with interpreting results.

subitem not at all important

1 ○

2 ○

3 ○

4 ○

5 ○

essential

Does your paper address subitem 5-ii?

Copy and paste relevant sections from the manuscript (include quotes in quotation marks "like this" to indicate direct quotes from your manuscript), or elaborate on this item by providing additional information not in the ms, or briefly explain why the item is not applicable/relevant for your study

Oma vastauksesi

Olet muokkaamassa vastaustasi. Jos jaat tämän URL-osoitteen, myös muut voivat muokata vastaustasi.

KIRJOITA UUSI VASTAUS

5-iii) Revisions and updating

Revisions and updating. Clearly mention the date and/or version number of the application/intervention (and comparator, if applicable) evaluated, or describe whether the intervention underwent major changes during the evaluation process, or whether the development and/or content was “frozen” during the trial. Describe dynamic components such as news feeds or changing content which may have an impact on the replicability of the intervention (for unexpected events see item 3b).

subitem not at all important

1 ○

2 ○

3 ○

4 ○

5 ○

essential

Does your paper address subitem 5-iii?

Copy and paste relevant sections from the manuscript (include quotes in quotation marks "like this" to indicate direct quotes from your manuscript), or elaborate on this item by providing additional information not in the ms, or briefly explain why the item is not applicable/relevant for your study

Oma vastauksesi

Olet muokkaamassa vastaustasi. Jos jaat tämän URL-osoitteen, myös muut voivat muokata vastaustasi.

KIRJOITA UUSI VASTAUS

5-iv) Quality assurance methods

Provide information on quality assurance methods to ensure accuracy and quality of information provided [1], if applicable.

subitem not at all important

1 ○

2 ○

3 ○

4 ○

5 ○

essential

Does your paper address subitem 5-iv?

Copy and paste relevant sections from the manuscript (include quotes in quotation marks "like this" to indicate direct quotes from your manuscript), or elaborate on this item by providing additional information not in the ms, or briefly explain why the item is not applicable/relevant for your study

Oma vastauksesi

Olet muokkaamassa vastaustasi. Jos jaat tämän URL-osoitteen, myös muut voivat muokata vastaustasi.

KIRJOITA UUSI VASTAUS

5-v) Ensure replicability by publishing the source code, and/or providing screenshots/screen-capture video, and/or providing flowcharts of the algorithms used

Ensure replicability by publishing the source code, and/or providing screenshots/screen-capture video, and/or providing flowcharts of the algorithms used. Replicability (i.e., other researchers should in principle be able to replicate the study) is a hallmark of scientific reporting.

subitem not at all important

1

2

3

4

5

essential

Does your paper address subitem 5-v?

Copy and paste relevant sections from the manuscript (include quotes in quotation marks "like this" to indicate direct quotes from your manuscript), or elaborate on this item by providing additional information not in the ms, or briefly explain why the item is not applicable/relevant for your study

Oma vastauksesi

Olet muokkaamassa vastaustasi. Jos jaat tämän URL-osoitteen, myös muut voivat muokata vastaustasi.

KIRJOITA UUSI VASTAUS

5-vi) Digital preservation

Digital preservation: Provide the URL of the application, but as the intervention is likely to change or disappear over the course of the years; also make sure the intervention is archived (Internet Archive, webcitation.org, and/or publishing the source code or screenshots/videos alongside the article). As pages behind login screens cannot be archived, consider creating demo pages which are accessible without login.

subitem not at all important

1 ○

2 ○

3 ○

4 ○

5 ○

essential

Does your paper address subitem 5-vi?

Copy and paste relevant sections from the manuscript (include quotes in quotation marks "like this" to indicate direct quotes from your manuscript), or elaborate on this item by providing additional information not in the ms, or briefly explain why the item is not applicable/relevant for your study

Oma vastauksesi

Olet muokkaamassa vastaustasi. Jos jaat tämän URL-osoitteen, myös muut voivat muokata vastaustasi.

KIRJOITA UUSI VASTAUS

5-vii) Access

Access: Describe how participants accessed the application, in what setting/context, if they had to pay (or were paid) or not, whether they had to be a member of specific group. If known, describe how participants obtained “access to the platform and Internet” [1]. To ensure access for editors/reviewers/readers, consider to provide a “backdoor” login account or demo mode for reviewers/readers to explore the application (also important for archiving purposes, see vi).

subitem not at all important

1 ○

2 ○

3 ○

4 ○

5 ○

essential

Does your paper address subitem 5-vii? *

Copy and paste relevant sections from the manuscript (include quotes in quotation marks "like this" to indicate direct quotes from your manuscript), or elaborate on this item by providing additional information not in the ms, or briefly explain why the item is not applicable/relevant for your study

Yes, a login for eligble patients was created by study nurse

Olet muokkaamassa vastaustasi. Jos jaat tämän URL-osoitteen, myös muut voivat muokata vastaustasi.

KIRJOITA UUSI VASTAUS

5-viii) Mode of delivery, features/functionalities/components of the intervention and comparator, and the theoretical framework

Describe mode of delivery, features/functionalities/components of the intervention and comparator, and the theoretical framework [6] used to design them (instructional strategy [1], behaviour change techniques, persuasive features, etc., see e.g., [7, 8] for terminology). This includes an in-depth description of the content (including where it is coming from and who developed it) [1],” whether [and how] it is tailored to individual circumstances and allows users to track their progress and receive feedback” [6]. This also includes a description of communication delivery channels and – if computer-mediated communication is a component – whether communication was synchronous or asynchronous [6]. It also includes information on presentation strategies [1], including page design principles, average amount of text on pages, presence of hyperlinks to other resources, etc. [1].

subitem not at all important

1 ○

2 ○

3 ○

4 ○

5 ○

essential

Olet muokkaamassa vastaustasi. Jos jaat tämän URL-osoitteen, myös muut voivat muokata vastaustasi.

KIRJOITA UUSI VASTAUS

Does your paper address subitem 5-viii? *

Copy and paste relevant sections from the manuscript (include quotes in quotation marks "like this" to indicate direct quotes from your manuscript), or elaborate on this item by providing additional information not in the ms, or briefly explain why the item is not applicable/relevant for your study

Yes, Noona® is a patient outcomes management solution designed to engage patients in their care with real-time symptom reporting and monitoring, The first module of the digital Noona solution was the module for follow-up of EBC and it was designed in collaboration with HUS. The symptom questionnaires and answers to the most common questions were designed according to the contents of the standard operational procedure (SOP) for breast cancer nurse practitioners on the follow-up of EBC.

In the first page of the questionnaire was listed and asked if the patient had any of the most common symptoms related to BC recurrence or adverse events of treatments. Additionally, there was an open question where the patient could report any other symptoms. If the patient reported having symptoms (any of the listed ones, or as free text) a further questionnaire related to the severity of the symptom according to the Common Terminology Criteria for Adverse Events (CTCAE) came into view. The Noona solution was designed in collaboration with senior experts in clinical breast oncology and IT solutions.

5-ix) Describe use parameters

Describe use parameters (e.g., intended “doses” and optimal timing for use). Clarify what instructions or recommendations were given to the user, e.g., regarding timing, frequency, heaviness of use, if any, or was the intervention used ad libitum.

subitem not at all important

1 ○

2 ○

3 ○

4 ○

5 ○

essential

Olet muokkaamassa vastaustasi. Jos jaat tämän URL-osoitteen, myös muut voivat muokata vastaustasi.

KIRJOITA UUSI VASTAUS

Does your paper address subitem 5-ix?

Copy and paste relevant sections from the manuscript (include quotes in quotation marks "like this" to indicate direct quotes from your manuscript), or elaborate on this item by providing additional information not in the ms, or briefly explain why the item is not applicable/relevant for your study

Oma vastauksesi

5-x) Clarify the level of human involvement

Clarify the level of human involvement (care providers or health professionals, also technical assistance) in the e-intervention or as co-intervention (detail number and expertise of professionals involved, if any, as well as "type of assistance offered, the timing and frequency of the support, how it is initiated, and the medium by which the assistance is delivered". It may be necessary to distinguish between the level of human involvement required for the trial, and the level of human involvement required for a routine application outside of a RCT setting (discuss under item 21 – generalizability).

subitem not at all important

1 ○

2 ○

3 ○

4 ○

5 ○

essential

Does your paper address subitem 5-x?

Copy and paste relevant sections from the manuscript (include quotes in quotation marks "like this" to indicate direct quotes from your manuscript), or elaborate on this item by providing additional information not in the ms, or briefly explain why the item is not applicable/relevant for your study

Oma vastauksesi

Olet muokkaamassa vastaustasi. Jos jaat tämän URL-osoitteen, myös muut voivat muokata vastaustasi.

KIRJOITA UUSI VASTAUS

5-xi) Report any prompts/reminders used

Report any prompts/reminders used: Clarify if there were prompts (letters, emails, phone calls, SMS) to use the application, what triggered them, frequency etc. It may be necessary to distinguish between the level of prompts/reminders required for the trial, and the level of prompts/reminders for a routine application outside of a RCT setting (discuss under item 21 – generalizability).

subitem not at all important

1 ○

2 ○

3 ○

4 ○

5 ○

essential

Does your paper address subitem 5-xi? *

Copy and paste relevant sections from the manuscript (include quotes in quotation marks "like this" to indicate direct quotes from your manuscript), or elaborate on this item by providing additional information not in the ms, or briefly explain why the item is not applicable/relevant for your study

Yes, reminders by study nurse if needed

Olet muokkaamassa vastaustasi. Jos jaat tämän URL-osoitteen, myös muut voivat muokata vastaustasi.

KIRJOITA UUSI VASTAUS

5-xii) Describe any co-interventions (incl. training/support)

Describe any co-interventions (incl. training/support): Clearly state any interventions that are provided in addition to the targeted eHealth intervention, as ehealth intervention may not be designed as stand-alone intervention. This includes training sessions and support [1]. It may be necessary to distinguish between the level of training required for the trial, and the level of training for a routine application outside of a RCT setting (discuss under item 21 – generalizability.

subitem not at all important

1 ○

2 ○

3 ○

4 ○

5 ○

essential

Does your paper address subitem 5-xii? *

Copy and paste relevant sections from the manuscript (include quotes in quotation marks "like this" to indicate direct quotes from your manuscript), or elaborate on this item by providing additional information not in the ms, or briefly explain why the item is not applicable/relevant for your study

Yes, Trial information was collected by paper. Intervention function was digital and can be found in Noona.

6a) Completely defined pre-specified primary and secondary outcome measures, including how and when they were assessed

Olet muokkaamassa vastaustasi. Jos jaat tämän URL-osoitteen, myös muut voivat muokata vastaustasi.

KIRJOITA UUSI VASTAUS

Does your paper address CONSORT subitem 6a? *

Copy and paste relevant sections from the manuscript (include quotes in quotation marks "like this" to indicate direct quotes from your manuscript), or elaborate on this item by providing additional information not in the ms, or briefly explain why the item is not applicable/relevant for your study

Yes, "The costs of specialized medical care were obtained from the Ecomed database of the Helsinki and Uusimaa Hospital District. Ecomed records contain all hospital resource use and costs including information about the exact day on which the hospital contact started and ended, diagnosis and procedure code, type of contact and patient level costs, including overheads, equipment, hospitalization, and drugs for inpatient use. Hospital contacts included hospitalization periods, emergency visits, doctor or nurse appointments, phone calls and letters, and rehabilitation. Contacts to the Department of oncology of the CCC were classified as contacts due to breast cancer.
Costs of out-patient contacts included out-patient appointments with a doctor or nurse, rehabilitation, medical aids (e.g., compression sleeves), letters and phone calls from hospital personnel to the patient.
The costs of phone calls to the nurse and use of the digital “Noona” solution were free from charge, and thus not included in the costs in the analysis. The definition of a letter and phone call is a pre-arranged individual letter or phone call about the diagnostics and treatment of the patient's illness, which can replace a visit.
Costs of diagnostics included laboratory tests, pathology reports, and imaging. " For other earlier published endpoints: "At 12 months the patients were asked in a written paper questionnaire if they preferred to use the digital Noona solution (Supplemental Table 1), telephone call service or both. Preference was analyzed as the primary endpoint after the patients had contacted the breast cancer nurse practitioner at least once with both modalities. The patients were also asked reasons for their preference with free text fields.

In addition, patients completed a written paper satisfaction survey at 6 and 12 months. The questionnaire for patient satisfaction was designed for the present study (Supplemental Table 1) since no validated questionnaires to address the purpose of the present study were available at the time of study design. The score of the questionnaire ranges between one and four. The overall satisfaction score was calculated as the mean of the scores for questions 1–15.

The health-related quality of life (HRQoL) was measured using The European Organization for Research and Treatment of Cancer Quality of Life Questionnaire Core30 questionnaire (EORTC QLQ-C30) [Citation12] with its breast cancer-specific module (EORTC QLQ-BR23) [Citation13] and the generic 15D which is a 15-dimensional questionnaire providing both a health profile and a preference-based single index score [Citation14]. The patients answered HRQoL questionnaires on paper at baseline and at 6 and 12 months thereafter. The questionnaires were on paper since digital versions were not available at the time of study design."

Olet muokkaamassa vastaustasi. Jos jaat tämän URL-osoitteen, myös muut voivat muokata vastaustasi.

KIRJOITA UUSI VASTAUS

6a-i) Online questionnaires: describe if they were validated for online use and apply CHERRIES items to describe how the questionnaires were designed/deployed

If outcomes were obtained through online questionnaires, describe if they were validated for online use and apply CHERRIES items to describe how the questionnaires were designed/deployed [9].

subitem not at all important

1

2

3

4

5

essential

Does your paper address subitem 6a-i?

Copy and paste relevant sections from manuscript text

Oma vastauksesi

Olet muokkaamassa vastaustasi. Jos jaat tämän URL-osoitteen, myös muut voivat muokata vastaustasi.

KIRJOITA UUSI VASTAUS

6a-ii) Describe whether and how “use” (including intensity of use/dosage) was defined/measured/monitored

Describe whether and how “use” (including intensity of use/dosage) was defined/measured/monitored (logins, logfile analysis, etc.). Use/adoption metrics are important process outcomes that should be reported in any ehealth trial.

subitem not at all important

1 ○

2 ○

3 ○

4 ○

5 ○

essential

Does your paper address subitem 6a-ii?

Copy and paste relevant sections from manuscript text

Oma vastauksesi

Olet muokkaamassa vastaustasi. Jos jaat tämän URL-osoitteen, myös muut voivat muokata vastaustasi.

KIRJOITA UUSI VASTAUS

6a-iii) Describe whether, how, and when qualitative feedback from participants was obtained

Describe whether, how, and when qualitative feedback from participants was obtained (e.g., through emails, feedback forms, interviews, focus groups).

subitem not at all important

1 ○

2 ○

3 ○

4 ○

5 ○

essential

Does your paper address subitem 6a-iii?

Copy and paste relevant sections from manuscript text

Oma vastauksesi

6b) Any changes to trial outcomes after the trial commenced, with reasons

Does your paper address CONSORT subitem 6b? *

Copy and paste relevant sections from the manuscript (include quotes in quotation marks "like this" to indicate direct quotes from your manuscript), or elaborate on this item by providing additional information not in the ms, or briefly explain why the item is not applicable/relevant for your study

No changes to trial outcomes
after the trial commenced was done

Olet muokkaamassa vastaustasi. Jos jaat tämän URL-osoitteen, myös muut voivat muokata vastaustasi. KIRJOITA UUSI VASTAUS

7a) How sample size was determined

NPT: When applicable, details of whether and how the clustering by care provides or centers was addressed

7a-i) Describe whether and how expected attrition was taken into account when calculating the sample size

Describe whether and how expected attrition was taken into account when calculating the sample size.

subitem not at all important

1 ○

2 ○

3 ○

4 ○

5 ○

essential

Does your paper address subitem 7a-i?

Copy and paste relevant sections from manuscript title (include quotes in quotation marks "like this" to indicate direct quotes from your manuscript), or elaborate on this item by providing additional information not in the ms, or briefly explain why the item is not applicable/relevant for your study

Oma vastauksesi

7b) When applicable, explanation of any interim analyses and stopping guidelines

Olet muokkaamassa vastaustasi. Jos jaat tämän URL-osoitteen, myös muut voivat muokata vastaustasi.

KIRJOITA UUSI VASTAUS

Does your paper address CONSORT subitem 7b? *

Copy and paste relevant sections from the manuscript (include quotes in quotation marks "like this" to indicate direct quotes from your manuscript), or elaborate on this item by providing additional information not in the ms, or briefly explain why the item is not applicable/relevant for your study

Not applicable, no intermin analyses

8a) Method used to generate the random allocation sequence

NPT: When applicable, how care providers were allocated to each trial group

Does your paper address CONSORT subitem 8a? *

Copy and paste relevant sections from the manuscript (include quotes in quotation marks "like this" to indicate direct quotes from your manuscript), or elaborate on this item by providing additional information not in the ms, or briefly explain why the item is not applicable/relevant for your study

Yes, At the final visit of adjuvant radiotherapy patients were recruited and randomized (with the method used in HUS at the time) to surveillance during the following year outside preplanned visits by means of standard phone calls or the interventional digital Noona solution.

8b) Type of randomisation; details of any restriction (such as blocking and block size)

Does your paper address CONSORT subitem 8b? *

Copy and paste relevant sections from the manuscript (include quotes in quotation marks "like this" to indicate direct quotes from your manuscript), or elaborate on this item by providing additional information not in the ms, or briefly explain why the item is not applicable/relevant for your study

Yes, the method used in HUS at the time

Olet muokkaamassa vastaustasi. Jos jaat tämän URL-osoitteen, myös muut voivat muokata vastaustasi.

KIRJOITA UUSI VASTAUS

9) Mechanism used to implement the random allocation sequence (such as sequentially numbered containers), describing any steps taken to conceal the sequence until interventions were assigned

Does your paper address CONSORT subitem 9? *

Copy and paste relevant sections from the manuscript (include quotes in quotation marks "like this" to indicate direct quotes from your manuscript), or elaborate on this item by providing additional information not in the ms, or briefly explain why the item is not applicable/relevant for your study

This was done according to GCP standards at the time

10) Who generated the random allocation sequence, who enrolled participants, and who assigned participants to interventions

Does your paper address CONSORT subitem 10? *

Copy and paste relevant sections from the manuscript (include quotes in quotation marks "like this" to indicate direct quotes from your manuscript), or elaborate on this item by providing additional information not in the ms, or briefly explain why the item is not applicable/relevant for your study

Yes, the study nurse did this

11a) If done, who was blinded after assignment to interventions (for example, participants, care providers, those assessing outcomes) and how

NPT: Whether or not administering co-interventions were blinded to group assignment

Olet muokkaamassa vastaustasi. Jos jaat tämän URL-osoitteen, myös muut voivat muokata vastaustasi.

KIRJOITA UUSI VASTAUS

11a-i) Specify who was blinded, and who wasn’t

Specify who was blinded, and who wasn’t. Usually, in web-based trials it is not possible to blind the participants [1, 3] (this should be clearly acknowledged), but it may be possible to blind outcome assessors, those doing data analysis or those administering co-interventions (if any).

subitem not at all important

1 ◯

2 ◯

3 ◯

4 ◯

5 ◯

essential

Does your paper address subitem 11a-i? *

Copy and paste relevant sections from the manuscript (include quotes in quotation marks "like this" to indicate direct quotes from your manuscript), or elaborate on this item by providing additional information not in the ms, or briefly explain why the item is not applicable/relevant for your study

This trial was not blinded

Olet muokkaamassa vastaustasi. Jos jaat tämän URL-osoitteen, myös muut voivat muokata vastaustasi.

KIRJOITA UUSI VASTAUS

11a-ii) Discuss e.g., whether participants knew which intervention was the "intervention of interest" and which one was the "comparator"

Informed consent procedures (4a-ii) can create biases and certain expectations - discuss e.g., whether participants knew which intervention was the "intervention of interest" and which one was the "comparator".

subitem not at all important

1 ○

2 ○

3 ○

4 ○

5 ○

essential

Does your paper address subitem 11a-ii?

Copy and paste relevant sections from the manuscript (include quotes in quotation marks "like this" to indicate direct quotes from your manuscript), or elaborate on this item by providing additional information not in the ms, or briefly explain why the item is not applicable/relevant for your study

Oma vastauksesi

11b) If relevant, description of the similarity of interventions

(this item is usually not relevant for ehealth trials as it refers to similarity of a placebo or sham intervention to a active medication/intervention)

Does your paper address CONSORT subitem 11b? *

Copy and paste relevant sections from the manuscript (include quotes in quotation marks "like this" to indicate direct quotes from your manuscript), or elaborate on this item by providing additional information not in the ms, or briefly explain why the item is not applicable/relevant for your study

Olet muokkaamassa vastaustasi. Jos jaat tämän URL-osoitteen, myös muut voivat muokata vastaustasi.

KIRJOITA UUSI VASTAUS

12a) Statistical methods used to compare groups for primary and secondary outcomes

NPT: When applicable, details of whether and how the clustering by care providers or centers was addressed

Does your paper address CONSORT subitem 12a? *

Copy and paste relevant sections from the manuscript (include quotes in quotation marks "like this" to indicate direct quotes from your manuscript), or elaborate on this item by providing additional information not in the ms, or briefly explain why the item is not applicable/relevant for your study

Yes, "The effect of the follow-up method was analyzed in repeated measurements general linear model analysis with follow-up method as repeated measurement variable and randomization group as a between-patients variable. This design allows testing the effect of both follow-up methods and the effect of timing (first or second 6-month period) on numbers of visits and costs. Dependent variables were number of visits, costs of diagnostics and visits, and total costs.

Descriptive statistics were calculated for the digital solution and the phone calls solution at baseline at six months and at 12 months.
" No imputation of missing values was used.

12a-i) Imputation techniques to deal with attrition / missing values

Imputation techniques to deal with attrition / missing values: Not all participants will use the intervention/comparator as intended and attrition is typically high in ehealth trials. Specify how participants who did not use the application or dropped out from the trial were treated in the statistical analysis (a complete case analysis is strongly discouraged, and simple imputation techniques such as LOCF may also be problematic [4]).

subitem not at all important

1 ○

2 ○

3 ○

4 ○

5 ○

Olet muokkaamassa vastaustasi. Jos jaat tämän URL-osoitteen, myös muut voivat muokata vastaustasi.

KIRJOITA UUSI VASTAUS

Does your paper address subitem 12a-i? *

Copy and paste relevant sections from the manuscript (include quotes in quotation marks "like this" to indicate direct quotes from your manuscript), or elaborate on this item by providing additional information not in the ms, or briefly explain why the item is not applicable/relevant for your study

Yes, no imputation of missing values was used

12b) Methods for additional analyses, such as subgroup analyses and adjusted analyses

Does your paper address CONSORT subitem 12b? *

Copy and paste relevant sections from the manuscript (include quotes in quotation marks "like this" to indicate direct quotes from your manuscript), or elaborate on this item by providing additional information not in the ms, or briefly explain why the item is not applicable/relevant for your study

Yes, "The effect of the follow-up method was analyzed in repeated measurements general linear model analysis with follow-up method as repeated measurement variable and randomization group as a between-patients variable. This design allows testing the effect of both follow-up methods and the effect of timing (first or second 6-month period) on numbers of visits and costs. Dependent variables were number of visits, costs of diagnostics and visits, and total costs.

Descriptive statistics were calculated for the digital solution and the phone calls solution at baseline at six months and at 12 months."

X26) REB/IRB Approval and Ethical Considerations [recommended as subheading under "Methods"] (not a CONSORT item)

Olet muokkaamassa vastaustasi. Jos jaat tämän URL-osoitteen, myös muut voivat muokata vastaustasi.

KIRJOITA UUSI VASTAUS

X26-i) Comment on ethics committee approval

subitem not at all important

1 ◯

2 ◯

3 ◯

4 ◯

5 ◯

essential

Does your paper address subitem X26-i?

Copy and paste relevant sections from the manuscript (include quotes in quotation marks "like this" to indicate direct quotes from your manuscript), or elaborate on this item by providing additional information not in the ms, or briefly explain why the item is not applicable/relevant for your study

Oma vastauksesi

x26-ii) Outline informed consent procedures

Outline informed consent procedures e.g., if consent was obtained offline or online (how? Checkbox, etc.?), and what information was provided (see 4a-ii). See [6] for some items to be included in informed consent documents.

subitem not at all important

1 ◯

2 ◯

3 ◯

4 ◯

Olet muokkaamassa vastaustasi. Jos jaat tämän URL-osoitteen, myös muut voivat muokata vastaustasi.

KIRJOITA UUSI VASTAUS

Does your paper address subitem X26-ii?

Copy and paste relevant sections from the manuscript (include quotes in quotation marks "like this" to indicate direct quotes from your manuscript), or elaborate on this item by providing additional information not in the ms, or briefly explain why the item is not applicable/relevant for your study

Oma vastauksesi

X26-iii) Safety and security procedures

Safety and security procedures, incl. privacy considerations, and any steps taken to reduce the likelihood or detection of harm (e.g., education and training, availability of a hotline)

subitem not at all important

1 ○

2 ○

3 ○

4 ○

5 ○

essential

Does your paper address subitem X26-iii?

Copy and paste relevant sections from the manuscript (include quotes in quotation marks "like this" to indicate direct quotes from your manuscript), or elaborate on this item by providing additional information not in the ms, or briefly explain why the item is not applicable/relevant for your study

Oma vastauksesi

RESULTS

Olet muokkaamassa vastaustasi. Jos jaat tämän URL-osoitteen, myös muut voivat muokata vastaustasi.

KIRJOITA UUSI VASTAUS

13a) For each group, the numbers of participants who were randomly assigned, received intended treatment, and were analysed for the primary outcome

NPT: The number of care providers or centers performing the intervention in each group and the number of patients treated by each care provider in each center

Does your paper address CONSORT subitem 13a? *

Copy and paste relevant sections from the manuscript (include quotes in quotation marks "like this" to indicate direct quotes from your manuscript), or elaborate on this item by providing additional information not in the ms, or briefly explain why the item is not applicable/relevant for your study

Yes, please see the flow chart in Figure 1

13b) For each group, losses and exclusions after randomisation, together with reasons

Does your paper address CONSORT subitem 13b? (NOTE: Preferably, this is shown in a CONSORT flow diagram) *

Copy and paste relevant sections from the manuscript (include quotes in quotation marks "like this" to indicate direct quotes from your manuscript), or elaborate on this item by providing additional information not in the ms, or briefly explain why the item is not applicable/relevant for your study

Yes, please see the flow chart in Figure 1

Olet muokkaamassa vastaustasi. Jos jaat tämän URL-osoitteen, myös muut voivat muokata vastaustasi.

KIRJOITA UUSI VASTAUS

13b-i) Attrition diagram

Strongly recommended: An attrition diagram (e.g., proportion of participants still logging in or using the intervention/comparator in each group plotted over time, similar to a survival curve) or other figures or tables demonstrating usage/dose/engagement.

subitem not at all important

1 ○

2 ○

3 ○

4 ○

5 ○

essential

Does your paper address subitem 13b-i?

Copy and paste relevant sections from the manuscript or cite the figure number if applicable (include quotes in quotation marks "like this" to indicate direct quotes from your manuscript), or elaborate on this item by providing additional information not in the ms, or briefly explain why the item is not applicable/relevant for your study

Oma vastauksesi

14a) Dates defining the periods of recruitment and follow-up

Does your paper address CONSORT subitem 14a? *

Copy and paste relevant sections from the manuscript (include quotes in quotation marks "like this" to indicate direct quotes from your manuscript), or elaborate on this item by providing additional information not in the ms, or briefly explain why the item is not applicable/relevant for your study

No such events were indicated

Olet muokkaamassa vastaustasi. Jos jaat tämän URL-osoitteen, myös muut voivat muokata vastaustasi.

KIRJOITA UUSI VASTAUS

14a-i) Indicate if critical “secular events” fell into the study period

Indicate if critical “secular events” fell into the study period, e.g., significant changes in Internet resources available or “changes in computer hardware or Internet delivery resources”

subitem not at all important

1

2

3

4

5

essential

Does your paper address subitem 14a-i?

Copy and paste relevant sections from the manuscript (include quotes in quotation marks "like this" to indicate direct quotes from your manuscript), or elaborate on this item by providing additional information not in the ms, or briefly explain why the item is not applicable/relevant for your study

Oma vastauksesi

14b) Why the trial ended or was stopped (early)

Does your paper address CONSORT subitem 14b? *

Copy and paste relevant sections from the manuscript (include quotes in quotation marks "like this" to indicate direct quotes from your manuscript), or elaborate on this item by providing additional information not in the ms, or briefly explain why the item is not applicable/relevant for your study

The trial was done as planned

Olet muokkaamassa vastaustasi. Jos jaat tämän URL-osoitteen, myös muut voivat muokata vastaustasi. KIRJOITA UUSI VASTAUS

15) A table showing baseline demographic and clinical characteristics for each group

NPT: When applicable, a description of care providers (case volume, qualification, expertise, etc.) and centers (volume) in each group

Does your paper address CONSORT subitem 15? *

Copy and paste relevant sections from the manuscript (include quotes in quotation marks "like this" to indicate direct quotes from your manuscript), or elaborate on this item by providing additional information not in the ms, or briefly explain why the item is not applicable/relevant for your study

Yes, Patient characteristics (n=734) are shown in Table 1.

15-i) Report demographics associated with digital divide issues

In ehealth trials it is particularly important to report demographics associated with digital divide issues, such as age, education, gender, social-economic status, computer/Internet/ehealth literacy of the participants, if known.

subitem not at all important

1 ○

2 ○

3 ○

4 ○

5 ○

essential

Does your paper address subitem 15-i? *

Copy and paste relevant sections from the manuscript (include quotes in quotation marks "like this" to indicate direct quotes from your manuscript), or elaborate on this item by providing additional information not in the ms, or briefly explain why the item is not applicable/relevant for your study

Olet muokkaamassa vastaustasi. Jos jaat tämän URL-osoitteen, myös muut voivat muokata vastaustasi.

KIRJOITA UUSI VASTAUS

16) For each group, number of participants (denominator) included in each analysis and whether the analysis was by original assigned groups

16-i) Report multiple “denominators” and provide definitions

Report multiple “denominators” and provide definitions: Report N’s (and effect sizes) “across a range of study participation [and use] thresholds” [1], e.g., N exposed, N consented, N used more than x times, N used more than y weeks, N participants “used” the intervention/comparator at specific pre-defined time points of interest (in absolute and relative numbers per group). Always clearly define “use” of the intervention.

subitem not at all important

1 ○

2 ○

3 ○

4 ○

5 ○

essential

Does your paper address subitem 16-i? *

Copy and paste relevant sections from the manuscript (include quotes in quotation marks "like this" to indicate direct quotes from your manuscript), or elaborate on this item by providing additional information not in the ms, or briefly explain why the item is not applicable/relevant for your study

Yes, these factors are reported in tables 2 and 3

Olet muokkaamassa vastaustasi. Jos jaat tämän URL-osoitteen, myös muut voivat muokata vastaustasi.

KIRJOITA UUSI VASTAUS

16-ii) Primary analysis should be intent-to-treat

Primary analysis should be intent-to-treat, secondary analyses could include comparing only “users”, with the appropriate caveats that this is no longer a randomized sample (see 18-i).

subitem not at all important

1 ○

2 ○

3 ○

4 ○

5 ○

essential

Does your paper address subitem 16-ii?

Copy and paste relevant sections from the manuscript (include quotes in quotation marks "like this" to indicate direct quotes from your manuscript), or elaborate on this item by providing additional information not in the ms, or briefly explain why the item is not applicable/relevant for your study

Oma vastauksesi

17a) For each primary and secondary outcome, results for each group, and the estimated effect size and its precision (such as 95% confidence interval)

Does your paper address CONSORT subitem 17a? *

Copy and paste relevant sections from the manuscript (include quotes in quotation marks "like this" to indicate direct quotes from your manuscript), or elaborate on this item by providing additional information not in the ms, or briefly explain why the item is not applicable/relevant for your study

Yes, these factors are reported in tables 2 and 3

Olet muokkaamassa vastaustasi. Jos jaat tämän URL-osoitteen, myös muut voivat muokata vastaustasi.

KIRJOITA UUSI VASTAUS

17a-i) Presentation of process outcomes such as metrics of use and intensity of use

In addition to primary/secondary (clinical) outcomes, the presentation of process outcomes such as metrics of use and intensity of use (dose, exposure) and their operational definitions is critical. This does not only refer to metrics of attrition (13-b) (often a binary variable), but also to more continuous exposure metrics such as “average session length”. These must be accompanied by a technical description how a metric like a “session” is defined (e.g., timeout after idle time) [1] (report under item 6a).

subitem not at all important

1 ○

2 ○

3 ○

4 ○

5 ○

essential

Does your paper address subitem 17a-i?

Copy and paste relevant sections from the manuscript (include quotes in quotation marks "like this" to indicate direct quotes from your manuscript), or elaborate on this item by providing additional information not in the ms, or briefly explain why the item is not applicable/relevant for your study

Oma vastauksesi

17b) For binary outcomes, presentation of both absolute and relative effect sizes is recommended

Olet muokkaamassa vastaustasi. Jos jaat tämän URL-osoitteen, myös muut voivat muokata vastaustasi.

KIRJOITA UUSI VASTAUS

Does your paper address CONSORT subitem 17b? *

Copy and paste relevant sections from the manuscript (include quotes in quotation marks "like this" to indicate direct quotes from your manuscript), or elaborate on this item by providing additional information not in the ms, or briefly explain why the item is not applicable/relevant for your study

Not applicable here in this study

18) Results of any other analyses performed, including subgroup analyses and adjusted analyses, distinguishing pre-specified from exploratory

Does your paper address CONSORT subitem 18? *

Copy and paste relevant sections from the manuscript (include quotes in quotation marks "like this" to indicate direct quotes from your manuscript), or elaborate on this item by providing additional information not in the ms, or briefly explain why the item is not applicable/relevant for your study

All the results are reported in results section

18-i) Subgroup analysis of comparing only users

A subgroup analysis of comparing only users is not uncommon in ehealth trials, but if done, it must be stressed that this is a self-selected sample and no longer an unbiased sample from a randomized trial (see 16-iii).

subitem not at all important

1 ◯

2 ◯

3 ◯

4 ◯

5 ◯

Olet muokkaamassa vastaustasi. Jos jaat tämän URL-osoitteen, myös muut voivat muokata vastaustasi.

KIRJOITA UUSI VASTAUS

Does your paper address subitem 18-i?

Copy and paste relevant sections from the manuscript (include quotes in quotation marks "like this" to indicate direct quotes from your manuscript), or elaborate on this item by providing additional information not in the ms, or briefly explain why the item is not applicable/relevant for your study

Oma vastauksesi

19) All important harms or unintended effects in each group
(for specific guidance see CONSORT for harms)

Does your paper address CONSORT subitem 19? *

Copy and paste relevant sections from the manuscript (include quotes in quotation marks "like this" to indicate direct quotes from your manuscript), or elaborate on this item by providing additional information not in the ms, or briefly explain why the item is not applicable/relevant for your study

Thi sis not applicable regarding this manuscript

19-i) Include privacy breaches, technical problems

Include privacy breaches, technical problems. This does not only include physical “harm” to participants, but also incidents such as perceived or real privacy breaches [1], technical problems, and other unexpected/unintended incidents. “Unintended effects” also includes unintended positive effects [2].

subitem not at all important

1 ○

2 ○

3 ○

4 ○

5 ○

Olet muokkaamassa vastaustasi. Jos jaat tämän URL-osoitteen, myös muut voivat muokata vastaustasi. KIRJOITA UUSI VASTAUS

Does your paper address subitem 19-i?

Copy and paste relevant sections from the manuscript (include quotes in quotation marks "like this" to indicate direct quotes from your manuscript), or elaborate on this item by providing additional information not in the ms, or briefly explain why the item is not applicable/relevant for your study

Oma vastauksesi

19-ii) Include qualitative feedback from participants or observations from staff/researchers

Include qualitative feedback from participants or observations from staff/researchers, if available, on strengths and shortcomings of the application, especially if they point to unintended/unexpected effects or uses. This includes (if available) reasons for why people did or did not use the application as intended by the developers.

subitem not at all important

1 ○

2 ○

3 ○

4 ○

5 ○

essential

Does your paper address subitem 19-ii?

Copy and paste relevant sections from the manuscript (include quotes in quotation marks "like this" to indicate direct quotes from your manuscript), or elaborate on this item by providing additional information not in the ms, or briefly explain why the item is not applicable/relevant for your study

Oma vastauksesi

Olet muokkaamassa vastaustasi. Jos jaat tämän URL-osoitteen, myös muut voivat muokata vastaustasi.

KIRJOITA UUSI VASTAUS

22) Interpretation consistent with results, balancing benefits and harms, and considering other relevant evidence

NPT: In addition, take into account the choice of the comparator, lack of or partial blinding, and unequal expertise of care providers or centers in each group

22-i) Restate study questions and summarize the answers suggested by the data, starting with primary outcomes and process outcomes (use)

Restate study questions and summarize the answers suggested by the data, starting with primary outcomes and process outcomes (use).

subitem not at all important

1 ○

2 ○

3 ○

4 ○

5 ○

essential

Does your paper address subitem 22-i? *

Copy and paste relevant sections from the manuscript (include quotes in quotation marks "like this" to indicate direct quotes from your manuscript), or elaborate on this item by providing additional information not in the ms, or briefly explain why the item is not applicable/relevant for your study

Yes, "The main finding of this study was that the number of hospital contacts and costs during the first follow-up year of early breast cancer patients with digital Noona solution did not differ from those of a traditional phone call service. During both surveillance methods the number of contacts and costs were higher during the latter 6 months of follow-up. This is probably explained by the pre-planned routine one year visit scheduled approximately 1 year after surgery, which for most patients occurred during the latter study period.
The mean number of hospital contacts at the department of oncology was 2.23 and the mean contact number was 4.50, when hospital contacts within other specialties than oncology were included. Thus, breast cancer survivors need frequent medical care both due to cancer and for other reasons during the early part of follow-up. The patients had e.g., [illegible]

Olet muokkaamassa vastaustasi. Jos jaat tämän URL-osoitteen, myös muut voivat muokata vastaustasi.

KIRJOITA UUSI VASTAUS

22-ii) Highlight unanswered new questions, suggest future research

Highlight unanswered new questions, suggest future research.

subitem not at all important

1
2
3
4
5

essential

Does your paper address subitem 22-ii?

Copy and paste relevant sections from the manuscript (include quotes in quotation marks "like this" to indicate direct quotes from your manuscript), or elaborate on this item by providing additional information not in the ms, or briefly explain why the item is not applicable/relevant for your study

Oma vastauksesi

20) Trial limitations, addressing sources of potential bias, imprecision, and, if relevant, multiplicity of analyses

Olet muokkaamassa vastaustasi. Jos jaat tämän URL-osoitteen, myös muut voivat muokata vastaustasi.

KIRJOITA UUSI VASTAUS

20-i) Typical limitations in ehealth trials

Typical limitations in ehealth trials: Participants in ehealth trials are rarely blinded. Ehealth trials often look at a multiplicity of outcomes, increasing risk for a Type I error. Discuss biases due to non-use of the intervention/usability issues, biases through informed consent procedures, unexpected events.

subitem not at all important

1 ○

2 ○

3 ○

4 ○

5 ○

essential

Does your paper address subitem 20-i? *

Copy and paste relevant sections from the manuscript (include quotes in quotation marks "like this" to indicate direct quotes from your manuscript), or elaborate on this item by providing additional information not in the ms, or briefly explain why the item is not applicable/relevant for your study

Yes, "We are aware of the limitations of the study. First, the study included the short follow-up time of 12 months, so long-term results could not be evaluated. Second, patients with relapsed disease were excluded from the study because of the crossover study design. Third, it is not possible to report the proportion of additional phone contacts during the digital solution period. Fourth, the data was collected from July 2015 to January 2017 and costs may have changed. Since the Covid-19 pandemic, the need for remote oncology services has expanded and the acceptability of using digital solutions may have increased. As the costs were the same, this should not have an impact on our results."

21) Generalisability (external validity, applicability) of the trial findings

NPT: External validity of the trial findings according to the intervention, comparators, patients, and care providers or centers involved in the trial

Olet muokkaamassa vastaustasi. Jos jaat tämän URL-osoitteen, myös muut voivat muokata vastaustasi.

KIRJOITA UUSI VASTAUS

21-i) Generalizability to other populations

Generalizability to other populations: In particular, discuss generalizability to a general Internet population, outside of a RCT setting, and general patient population, including applicability of the study results for other organizations

subitem not at all important

1 ◯

2 ◯

3 ◯

4 ◯

5 ◯

essential

Does your paper address subitem 21-i?

Copy and paste relevant sections from the manuscript (include quotes in quotation marks "like this" to indicate direct quotes from your manuscript), or elaborate on this item by providing additional information not in the ms, or briefly explain why the item is not applicable/relevant for your study

Oma vastauksesi

Olet muokkaamassa vastaustasi. Jos jaat tämän URL-osoitteen, myös muut voivat muokata vastaustasi. KIRJOITA UUSI VASTAUS

21-ii) Discuss if there were elements in the RCT that would be different in a routine application setting

Discuss if there were elements in the RCT that would be different in a routine application setting (e.g., prompts/reminders, more human involvement, training sessions or other co-interventions) and what impact the omission of these elements could have on use, adoption, or outcomes if the intervention is applied outside of a RCT setting.

subitem not at all important

1 ○

2 ○

3 ○

4 ○

5 ○

essential

Does your paper address subitem 21-ii?

Copy and paste relevant sections from the manuscript (include quotes in quotation marks "like this" to indicate direct quotes from your manuscript), or elaborate on this item by providing additional information not in the ms, or briefly explain why the item is not applicable/relevant for your study

Oma vastauksesi

OTHER INFORMATION

23) Registration number and name of trial registry

Olet muokkaamassa vastaustasi. Jos jaat tämän URL-osoitteen, myös muut voivat muokata vastaustasi.

KIRJOITA UUSI VASTAUS

Does your paper address CONSORT subitem 23? *

Copy and paste relevant sections from the manuscript (include quotes in quotation marks "like this" to indicate direct quotes from your manuscript), or elaborate on this item by providing additional information not in the ms, or briefly explain why the item is not applicable/relevant for your study

Yes, Trial Registration: ClinicalTrials.gov number: NCT04980989

24) Where the full trial protocol can be accessed, if available

Does your paper address CONSORT subitem 24? *

Cite a Multimedia Appendix, other reference, or copy and paste relevant sections from the manuscript (include quotes in quotation marks "like this" to indicate direct quotes from your manuscript), or elaborate on this item by providing additional information not in the ms, or briefly explain why the item is not applicable/relevant for your study

Yes, by contacting PI Johanna Mattson

25) Sources of funding and other support (such as supply of drugs), role of funders

Does your paper address CONSORT subitem 25? *

Copy and paste relevant sections from the manuscript (include quotes in quotation marks "like this" to indicate direct quotes from your manuscript), or elaborate on this item by providing additional information not in the ms, or briefly explain why the item is not applicable/relevant for your study

Yes, This work was partially funded by Helsinki University Hospital (HUS) and Noona Healthcare (no grant number) without any role in study design; in the collection, analysis and interpretation of data; in the writing of the report; and in the decision to submit the article for publication. This work was supported by Helsingin ja Uudenmaan Sairaanhoitopiiri.

X27) Conflicts of Interest (not a CONSORT item)

Olet muokkaamassa vastaustasi. Jos jaat tämän URL-osoitteen, myös muut voivat muokata vastaustasi.

KIRJOITA UUSI VASTAUS

X27-i) State the relation of the study team towards the system being evaluated

In addition to the usual declaration of interests (financial or otherwise), also state the relation of the study team towards the system being evaluated, i.e., state if the authors/evaluators are distinct from or identical with the developers/sponsors of the intervention.

subitem not at all important

1 ○

2 ○

3 ○

4 ○

5 ○

essential

Does your paper address subitem X27-i?

Copy and paste relevant sections from the manuscript (include quotes in quotation marks "like this" to indicate direct quotes from your manuscript), or elaborate on this item by providing additional information not in the ms, or briefly explain why the item is not applicable/relevant for your study

Oma vastauksesi

About the CONSORT EHEALTH checklist

As a result of using this checklist, did you make changes in your manuscript? *

○ yes, major changes

○ yes, minor changes

◉ no

Olet muokkaamassa vastaustasi. Jos jaat tämän URL-osoitteen, myös muut voivat muokata vastaustasi.

KIRJOITA UUSI VASTAUS

What were the most important changes you made as a result of using this checklist?

Oma vastauksesi

How much time did you spend on going through the checklist INCLUDING making changes in your manuscript *

I spent 2 hours in total,

As a result of using this checklist, do you think your manuscript has improved? *

- ○ yes
- ◉ no
- ○ Muu:

Would you like to become involved in the CONSORT EHEALTH group?

This would involve for example becoming involved in participating in a workshop and writing an "Explanation and Elaboration" document

- ○ yes
- ○ no
- ○ Muu:

Any other comments or questions on CONSORT EHEALTH

Oma vastauksesi

Olet muokkaamassa vastaustasi. Jos jaat tämän URL-osoitteen, myös muut voivat muokata vastaustasi.

KIRJOITA UUSI VASTAUS

STOP - Save this form as PDF before you click submit

To generate a record that you filled in this form, we recommend to generate a PDF of this page (on a Mac, simply select "print" and then select "print as PDF") before you submit it.

When you submit your (revised) paper to JMIR, please upload the PDF as supplementary file.

Don't worry if some text in the textboxes is cut off, as we still have the complete information in our database. Thank you!

Final step: Click submit !

Click submit so we have your answers in our database!

Lähetä

Älä koskaan lähetä salasanaa Google Formsin kautta.

Google ei ole luonut tai hyväksynyt tätä sisältöä. - Ota yhteyttä lomakkeen omistajaan - Käyttöehdot - Tietosuojakäytäntö

Näyttääkö lomake epäilyttävältä? Ilmoita

Google Forms

Olet muokkaamassa vastaustasi. Jos jaat tämän URL-osoitteen, myös muut voivat muokata vastaustasi.

KIRJOITA UUSI VASTAUS

Olet muokkaamassa vastaustasi. Jos jaat tämän URL-osoitteen, myös muut voivat muokata vastaustasi.

KIRJOITA UUSI VASTAUS
